# Association between polypharmacy and depression relapse in individuals with comorbid depression and type 2 diabetes: a UK electronic health record study

**DOI:** 10.1192/bjp.2022.160

**Published:** 2023-03

**Authors:** Annie Jeffery, Cini Bhanu, Kate Walters, Ian C. K. Wong, David Osborn, Joseph F. Hayes

**Affiliations:** Epidemiology and Applied Clinical Research Department, Division of Psychiatry, University College London (UCL), UK; Department of Primary Care & Population Health, Institute of Epidemiology & Health, University College London (UCL), London, UK; Research Department of Practice and Policy, School of Pharmacy, University College London (UCL), UK and Centre for Safe Medication Practice and Research, Department of Pharmacology and Pharmacy, Li Ka Shing Faculty of Medicine, The University of Hong Kong, Hong Kong

**Keywords:** Antidepressants, depressive disorders, comorbidity, polypharmacy, epidemiology

## Abstract

**Background:**

Individuals with physical comorbidities and polypharmacy may be at higher risk of depression relapse, however, they are not included in the ‘high risk of relapse’ group for whom longer antidepressant treatment durations are recommended.

**Aims:**

In individuals with comorbid depression and type 2 diabetes (T2DM), we aimed to investigate the association and interaction between depression relapse and (a) polypharmacy, (b) previous duration of antidepressant treatment.

**Method:**

This was a cohort study using primary care data from the UK Clinical Practice Research Datalink (CPRD) from years 2000 to 2018. We used Cox regression models with penalised B-splines to describe the association between restarting antidepressants and our two exposures.

**Results:**

We identified 48 001 individuals with comorbid depression and T2DM, who started and discontinued antidepressant treatment during follow-up. Within 1 year of antidepressant discontinuation, 35% of participants restarted treatment indicating depression relapse. As polypharmacy increased, the rate of restarting antidepressants increased until a maximum of 18 concurrent medications, where individuals were more than twice as likely to restart antidepressants (hazard ratio (HR) = 2.15, 95% CI 1.32–3.51). As the duration of previous antidepressant treatment increased, the rate of restarting antidepressants increased – individuals with a previous duration of ≥25 months were more than twice as likely to restart antidepressants than those who previously discontinued in <7 months (HR = 2.36, 95% CI 2.25–2.48). We found no interaction between polypharmacy and previous antidepressant duration.

**Conclusions:**

Polypharmacy and longer durations of previous antidepressant treatment may be associated with depression relapse following the discontinuation of antidepressant treatment.

## Background

Depression and type 2 diabetes mellitus (T2DM) are common conditions and both are major contributors to the global burden of disease.^1^ There is substantial evidence showing a bidirectional relationship between depression and T2DM,^2^ and between depression and worse outcomes in T2DM.^3^ Thus, the successful treatment of each condition is important for the management of the other.

The UK National Institute for Health and Care Excellence (NICE) recommend antidepressants as treatment for people with moderate-to-severe depression and physical comorbidities,^4^ and a Cochrane review of randomised-controlled trials (RCTs) has found evidence in the short term of a moderate improvement in depression symptoms for individuals with T2DM treated with antidepressants.^5^ Depression is often chronic, with many patients experiencing multiple relapses of depression after initially recovering from an episode.^6–9^ However, there are no previous studies that investigate longer-term outcomes, including depression relapse, in individuals with T2DM following the discontinuation of antidepressant treatment.^10^ Therefore, understanding factors associated with depression relapse in individuals with T2DM is an unmet research need.

## Polypharmacy

Evidence shows that the risk of depression relapse during the course of antidepressant treatment is increased in individuals with a higher burden from physical comorbidities.^7^ One type of burden from multimorbidity can come from the use of multiple medications, or ‘polypharmacy’. Polypharmacy has been shown to be associated with increased depression symptoms,^11^ and therefore may place individuals at higher risk of depression relapse because of the burden of living with multiple long-term conditions and taking complex medication regimens,^12^ or the potential adverse effects of the medications themselves and interactions between them.^13^ Polypharmacy is common in individuals with T2DM, with the need to control blood sugar levels, blood pressure and cholesterol in most individuals, as well as the prevention and management of potentially numerous complications.^14^

## Treatment duration

In the general population, individuals who discontinue antidepressants are more likely to relapse than those who remain on antidepressant treatment.^8,9^ However, what remains unclear from these reviews is the optimum duration of treatment, or the patient characteristics associated with relapse.

In order for antidepressants to be effective NICE guidelines recommend a minimum treatment duration of 6 months following the resolution of symptoms,^4^ however, international guidelines from the World Health Organization (WHO) recommend a minimum duration following the resolution of symptoms of 9–12 months.^15^ Both guidelines recommend maintenance therapy of at least 24 months for participants at higher risk of relapse, however, despite physical conditions such as T2DM^2^ and high levels of polypharmacy^11^ being associated with higher rates of depression, individuals with these characteristics are explicitly not included in the high risk of relapse group for which a longer treatment duration is recommended, although the definition of this group is unclear.

## Aims

Measuring polypharmacy and optimal real-world treatment durations is difficult in RCTs, which have strict inclusion criteria and defined end-points. On the other hand, depression relapse is difficult to measure using routine data, where individuals may or may not present to healthcare services and outcomes of interest such as depression symptoms may not be recorded systematically. Therefore, using electronic health record (EHR) data from UK primary care, we aimed to investigate restarting antidepressant treatment, following discontinuation, as a marker of clinically identified depression relapse in adults with comorbid depression and T2DM. Specifically, we aimed to describe:

- the proportion of individuals who restarted antidepressant treatment within the first year following discontinuation;
- the association between the number of concurrent medications prescribed before discontinuation and subsequently restarting antidepressant treatment;
- the association between the previous duration of antidepressant treatment and subsequently restarting antidepressant treatment;
- the interaction between the number of concurrent medications prescribed and the duration of previous antidepressant treatment, with regards to restarting antidepressant treatment.

We hypothesised that higher levels of polypharmacy would be associated with higher rates of restarting antidepressant treatment because of its association with increased depression symptoms. We hypothesised that shorter antidepressant treatment durations would be associated with higher rates of restarting antidepressant treatment because of the evidence of higher rates of relapse in individuals who discontinued rather than maintained antidepressant treatment. Additionally, we hypothesised that longer durations of previous antidepressant treatment would attenuate the association between higher polypharmacy levels and higher rates of restarting antidepressant treatment.

## Method

### Study design and setting

We carried out a cohort study using data from the UK Clinical Practice Research Datalink (CPRD) – a longitudinal data-set containing EHRs for over 60 million people, across 2000 primary care practices in the UK.^16^ The CPRD includes two separate databases that we combined: CPRD Gold and CPRD Aurum, each based on different computer software packages used for the EHRs. The data-sets are similar and include all demographic information, diagnoses, symptoms, laboratory tests and other health indicators recorded by the general practitioner, as well as all prescriptions issued. The CPRD has been shown to be representative of the UK population with respect to age, gender and ethnicity.^17,18^

Our study period ran from 1 January 2000 to 31 December 2018, however, we used data prior to the year 2000 for cohort selection and covariate identification.

### Ethics and consent statement

The authors assert that all procedures contributing to this work comply with the ethical standards of the relevant national and institutional committees on human experimentation and with the Helsinki Declaration of 1975, as revised in 2008. All procedures involving human patients were approved by the Independent Scientific Advisory Committee of CPRD (protocol no. 21_001648). All data sent to the CPRD is anonymised and therefore consent is not required.

### Participants

**Fig. 1:**
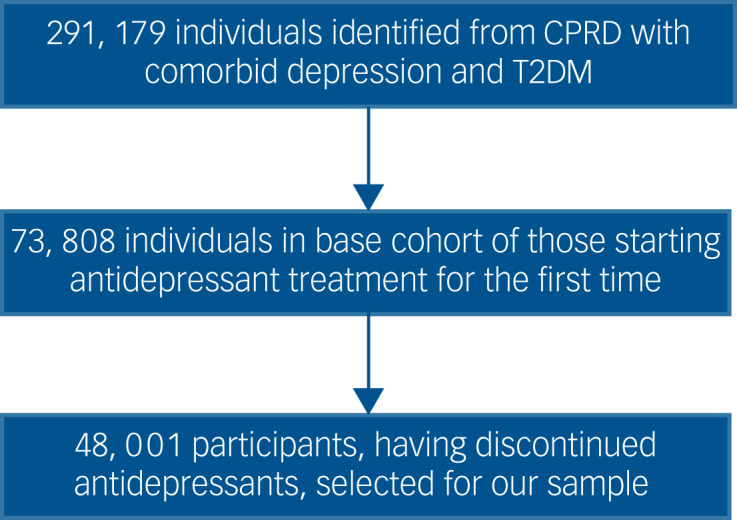
Participants included in the study. CPRD, Clinical Practice Research Datalink; T2DM, type 2 diabetes.

A flow chart of participant selection is shown in Fig. 1.

Our sample was taken from a cohort of individuals with comorbid depression and T2DM who had started antidepressant treatment for the first time within the study period and their EHR follow-up. This cohort was used to ensure we were able to calculate the duration of participants’ previous antidepressant use. Participants in the cohort met the following three inclusion criteria.

- *Depression*. Including: individuals with a clinical code for depression symptom, diagnosis or process of care; in preliminary work on defining the cohort we found that approximately 8.51% of individuals included in the CPRD met these criteria for depression and this is similar to the English national prevalence of 8.38%.^19^ Excluding: individuals who only had depression codes related to dementia, maternity, schizophrenia or bipolar disorder.
- *T2DM*. Including: individuals with at least two blood/serum glucose/haemoglobin A1c tests recorded above the threshold for T2DM and either a clinical code for T2DM or at least one antidiabetic medication prescription code; these inclusion criteria are based on previous research that shows the necessity of cross-validation for T2DM identification in EHRs.^20^ Excluding: individuals with clinical codes for type 1 diabetes mellitus, individuals with <6 months between the date of the first recorded oral antidiabetic prescription and the first recorded insulin prescription (possible type 1 or gestational diabetes), or individuals who only had codes or medication for T2DM present during periods of pregnancy (possible gestational diabetes only).
- *First antidepressant treatment during follow-up*. Including: individuals whose first recorded antidepressant prescription was monotherapy with a common first-line antidepressant (citalopram, escitalopram, fluoxetine, mirtazapine, paroxetine, sertraline, venlafaxine); we limited our inclusion criteria to these antidepressants as they are the most commonly prescribed antidepressants for the initial treatment of depression in the UK.^21^ Furthermore, as the clinical indication for prescription of antidepressants is not recorded in CPRD data we could not be confident that other antidepressants were being prescribed to treat depression – particularly those commonly prescribed for diabetic neuropathic pain, such as duloxetine. Excluding: individuals with <6 months of antidepressant-free data before the date of their first antidepressant prescription, to ensure that we were identifying incident (new-onset) prescribing; individuals whose first recorded antidepressant was an agent not listed in the inclusion criteria above, as these are uncommonly prescribed or used for other non-mental health conditions such as diabetic neuropathic pain.

Individuals entered this cohort when they commenced antidepressant treatment, which we considered to signify a clinically identified episode of depression. From this cohort, we included in our sample participants who discontinued antidepressant treatment between the years 2000 to 2018 and during their EHR follow-up. We defined antidepressant discontinuation as the absence of a recorded prescription for any other antidepressant within the first 60 days after expected duration of the last antidepressant prescription. We calculated the expected duration of the last antidepressant prescription using data on the number of tablets issued, recorded frequency and/or duration, length of previous prescriptions, or, where none of these were available, the median duration of 28 days. We specified a gap of 60 days to account for individuals who may collect their prescription at irregular intervals or who may restart antidepressant treatment within this period, because of withdrawal phenomena. Participants with <60 days follow-up after the expected duration of the final antidepressant before discontinuation were excluded.

### Outcomes

Our outcome was restarting antidepressant treatment within 365 days after the expected end date of the last antidepressant prescription before discontinuation. We specified a period 365 days of interest in line with other studies investigating depression relapse following antidepressant discontinuation in a UK primary care population.^8^

We aimed to investigate restarting antidepressant treatment as a marker of depression relapse, by using a participant-inclusion criteria that defined antidepressant discontinuation at ≥60 antidepressant-free days. We considered restarting antidepressant treatment following previous discontinuation as a proxy measure for depression relapse, because the clinician and patient have identified a need to restart treatment. Furthermore, all participants had previously sought help for depression from primary care and received antidepressants.

We censored participants after 365 days, at the date of death, the date that their primary care practice registration ended or 31 December 2018, whichever was sooner.

### Exposure

Our primary exposure was the number of concurrent medications prescribed simultaneous to the date of the last antidepressant prescription prior to discontinuation, or up to 90 days prior. This count did not include the antidepressant itself. We specified 90 days to allow for prescriptions issued for longer durations that may be relevant for chronic conditions and is in line with previous research defining polypharmacy.^22^ We included only pharmaceutical medications, and excluded topical medications, supplements and vaccinations.

Our secondary exposure was the duration of the initial course of antidepressant treatment prior to discontinuation. We measured this as the count in days between the first antidepressant prescription and the final antidepressant prescription before discontinuation, plus the expected duration of the final prescription. We categorised the duration of the initial antidepressant treatment into five categories, comparing different guideline recommendations, related to international treatment guidelines.

- Early discontinuation: 0–6 months.
- NICE recommendations: 7–9 months.
- WHO recommendations: 10–13 months.
- Medium-term: 14–24 months.
- NICE + WHO recommended maintenance for high-risk patients: ≥25 months.

We used the early discontinuation category as the reference category.

### Confounders

We included calendar year, age, gender, ethnicity and primary care practice as potential confounders. Where ethnicity was missing, we recoded this as ‘White’ – as the CPRD population has been found to be representative of the UK population in terms of ethnicity,^17,18^ 93% of more individuals with missing ethnicity would be expected to be of White ethnicity; this approach is in line with other research studies using the CPRD.^23^ We did not include comorbidities or diabetes treatment stage because of collinearity with our main exposure of polypharmacy.

### Sensitivity analyses

We performed three sensitivity analyses. The first in order to differentiate individuals with higher numbers of repeat (ongoing concurrent medication use) prescriptions compared with one-off concurrent prescriptions, we performed a sensitivity analysis redefining the exposure variable (the number of concurrent medications prescribed simultaneously to the date of the last antidepressant prescription before discontinuation) to include only repeat prescriptions, by requiring at least two prescriptions within 180 days prior to the index date, with at least one of these being within 90 days.

In order to investigate the effect of coding participants with missing ethnicity as ‘White’, we performed a sensitivity analysis, repeating our main analysis with ‘complete cases’ only.

In order to assess the role of timing for depression relapse (within 365 days of discontinuation), we performed a sensitivity analysis, whereby we limited the observed period to 182 days. As some studies define relapse as occurring within 6 months of discontinuation.^6^

### Statistical analyses

We performed two univariable analyses to estimate the association between restarting antidepressant treatment and each of our exposures separately (the number of concurrent medications, and the previous duration of antidepressant treatment). In our first multivariable model, we investigated the association between restarting antidepressant treatment and the number of concurrent medications, adjusted for the previous duration of antidepressant treatment and demographic confounders. In our second multivariable model, we investigated the association between restarting antidepressant treatment and the previous duration of antidepressant treatment, adjusted for demographic confounders. In both multivariable models, we included the primary care practice as a strata term, whereby separate baseline hazard functions are fitted for each strata to account for the clustering effect of each primary care practice.

We used Cox regression to investigate the association between restarting antidepressant treatment and our exposures. As a result of the non-linear relationship between antidepressant prescribing patterns and the number of concurrent medications, we used a penalised B-splines term to model the number of concurrent medications. This method enables the use of the linear Cox proportional hazards model, by fitting a number of linear functions to a non-linear relationship, to provide interval estimates. B-splines are piece-wise defined and therefore ideal to model and interpret integer exposures as they overcome numerical instability. The penalised fit balances flexibility against overfitting to define the optimal point for each spline at which the interval estimates are made. Spline points are automatically identified and assigned by this model, where an association between the exposure and outcome exists.

Finally, we modelled and examined interaction terms between the number of concurrent medications and the previous duration of antidepressant treatment with regards to restarting antidepressant treatment. We repeated the multivariable model for our sensitivity analysis with the redefined exposure variable for ongoing co-prescriptions.

All analysis was performed using R version 4.0.5.

## Results

From the cohort of adults with comorbid depression and T2DM who started antidepressant treatment for the first time we identified 48 001 participants who subsequently discontinued antidepressant treatment during the study period. A flow chart of participant inclusion is shown in Fig. 1.

The median age of participants was 62 (interquartile range (IQR) 54–71) and 53% were female. Full participant characteristics are described in Table 1. The median number of medications prescribed simultaneously to the date of the last antidepressant prescription prior to discontinuation, excluding the antidepressant itself, was nine, with six of those identified as repeat prescriptions.

**Table 1:** Participant characteristics and descriptive analysis

Characteristic	Value
Participants, *n*	48 001
Age, median (IQR)	62 (54–71)
Female, *n* (%)	25 437 (52.99)
Ethnicity group, *n* (%)	
Asian	3322 (6.92)
Black	1388 (2.89)
Mixed	262 (0.55)
Missing (imputed as White)	17 826 (37.14)
Other	357 (0.74)
White	24 846 (51.76)
Number of primary care practices included, *n*	1482
Number of co-prescriptions at baseline, median (IQR)	9 (6–12)
Number of ongoing co-prescriptions at baseline, median (IQR)	6 (4–9)
Previous antidepressant duration recommendations category, *n* (%)	
Early discontinuation (<7 months)	33 039 (68.83)
NICE (7–9 months^a^)	3167 (6.60)
WHO (10–13 months^a^)	2204 (4.59)
Medium-term (14–24 months^a^)	4370 (9.10)
Maintenance (≥25 months)	5221 (10.88)
Restarting antidepressant treatment	16 986 (35.39)
Censored	1560 (3.25)

a. Recommended initial duration of antidepressant treatment from NICE and WHO guidelines. IQR, interquartile range; NICE, National Institute for Health and Care Excellence; WHO, World Health Organization.

The majority of participants discontinued antidepressant treatment early with the median treatment duration of the first antidepressant prescribed at 2.79 months. Within the first year following discontinuation 35.39% of participants restarted antidepressant treatment.

Full results for our main analyses our given in Table 2 and for our sensitivity analyses in the Supplementary Tables 1 and 2 available at https://doi.org/10.1192/bjp.2022.160.

**Table 2:** Univariable and multivariable analysis for the association between primary and secondary exposures, and restarting antidepressant treatment

	Univariable HR (95% CI)	Multivariable Model 1^a^ HR (95% CI)	Multivariable Model 2^a^ HR (95% CI)
Number of medications^b^			
0	Reference point	Reference point	–
2	1.01 (1.01–1.14)	1.08 (1.01–1.15)	–
3	1.15 (1.04–1.27)	1.16 (1.05–1.29)	–
4	1.22 (1.09–1.37)	1.24 (1.10–1.40)	–
5	1.29 (1.14–1.45)	1.33 (1.17–1.50)	–
6	1.34 (1.20–1.51)	1.40 (1.23–1.58)	–
7	1.39 (1.24–1.57)	1.46 (1.29–1.66)	–
8	1.45 (1.29–1.63)	1.54 (1.36–1.75)	–
9	1.52 (1.35–1.71)	1.64 (1.44–1.86)	–
10	1.60 (1.40–1.79)	1.72 (1.51–1.95)	–
11	1.63 (1.44–1.86)	1.78 (1.55–2.04)	–
12	1.68 (1.46–1.94)	1.83 (1.58–2.12)	–
13	1.74 (1.47–2.05)	1.87 (1.58–2.22)	–
14	1.79 (1.46–2.19)	1.92 (1.56–2.36)	–
15	1.85 (1.44–2.38)	1.97 (1.52–2.56)	–
16	1.90 (1.39–2.62)	2.03 (1.47–2.81)	–
17	1.97 (1.33–2.93)	2.09 (1.40–3.12)	–
18	2.04 (1.26–3.31)	2.15 (1.32–3.51)	–
Duration of previous antidepressant			
<7 months	Reference category	–	Reference category
7–9 months	1.56 (1.48–1.66)	–	1.60 (1.51–1.70)
10–13 months	1.73 (1.62–1.84)	–	1.79 (1.68–1.92)
14–24 months	2.02 (1.93–2.12)	–	2.13 (2.03–2.23)
≥25 months	2.14 (2.04–2.24)	–	2.36 (2.25–2.48)

a. Multivariable model 1 estimated the association between restarting antidepressants and the number of concurrent medications, adjusted for the duration of previous antidepressant treatment, age, gender, ethnicity, calendar year and general practice. Multivariable model 2 estimated the association between restarting antidepressants and the duration of previous antidepressant treatment, adjusted for age, gender, ethnicity, calendar year and general practice.

b. Medications prescribed simultaneous to the date of the last antidepressant prescription before discontinuation, excluding the antidepressant itself.

**Fig. 2:**
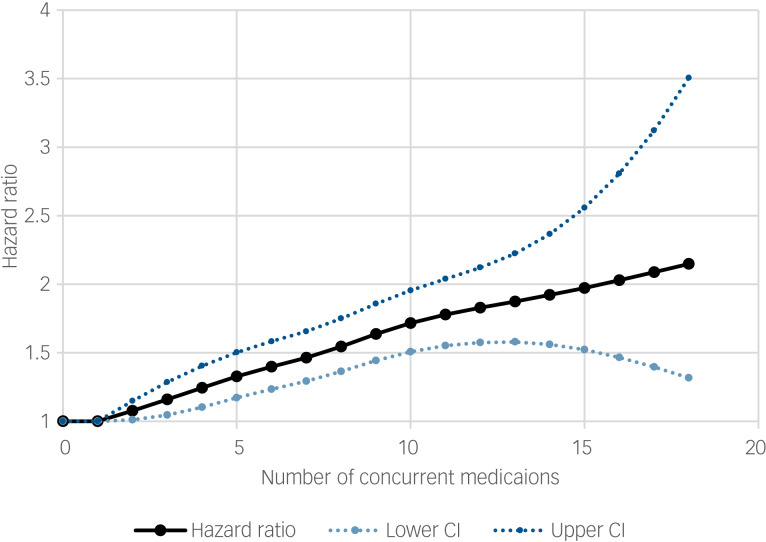
Hazard ratios for the changing rate of restarting antidepressant treatment, by the number of concurrent medications prescribed at the time of prior antidepressant discontinuation (adjusted for duration of previous antidepressant treatment, age, gender, ethnicity, calendar year and primary care practices). Reference group, 0 concurrent medications. CI = confidence interval.

In both the adjusted and unadjusted models, we found a dose–response relationship between the number of concurrent medications prescribed at the time of antidepressant discontinuation, and the rate of restarting antidepressant treatment (Fig. 2). The association began at two concurrent medications, with the rate of restarting antidepressant treatment following a linear increase for each additional medication prescribed, until 18 concurrent medications, at which point the maximum association was reached. The median number of nine concurrent medications was associated with a 64% increase in the rate of restarting (hazard ratio (HR) = 1.64, 95% CI 1.44–1.86). Our model did not fit a spline point before 1 concurrent medication or after 18 concurrent medications, indicating that there was no association between the number of concurrent medications and restarting antidepressants outside these points.

Adjustment for confounders did not significantly change the effect sizes in any of our multivariable models.

In our sensitivity analysis, which redefined the primary exposure variable to include repeat prescriptions only, the association between number of concurrent medications and restarting antidepressant treatment began at five repeat prescriptions and increased until 10 repeat prescriptions. The median number of six concurrent medications was associated with a 52% increase in the rate of restarting (HR = 1.52, 95% CI 1.11–2.08).

In all both the adjusted and unadjusted models, we found a dose–response relationship whereby the rate of restarting antidepressant treatment increased as the previous duration of antidepressant treatment increased. In our second multivariable model, individuals with previous durations of 7–9 months were 60% more likely to restart antidepressant treatment compared with individuals with previous durations of 1–6 months (HR = 1.60, 95% CI 1.51–1.70); at 9–12 months this increased to 79% (HR = 1.79, 95% CI 1.68–1.92); at 12–23 months to 113% (HR = 2.13, 95% CI 2.03–2.23); and at ≥24 months to 136% (HR = 2.36, 95% CI 2.25–2.48).

We found no evidence of an interaction between the number of concurrent medications prescribed at antidepressant discontinuation and the previous duration of antidepressant treatment, with regards to restarting antidepressant treatment (*P* > 0.05). We found no further differences in our sensitivity analyses.

## Discussion

### Main findings

This is the first study, to our knowledge, to investigate restarting antidepressant treatment following discontinuation in adults with comorbid depression and T2DM. We found that just over a third of participants (35%) restarted antidepressant treatment within the first year of discontinuation.

### Interpretation of our findings

We had hypothesised that higher levels of polypharmacy would be associated with an increased rate of restarting antidepressant treatment following discontinuation. Indeed, as the number of concurrent medications increased, the rate of restarting antidepressants also increased considerably, up to 115%. These results are in line with evidence showing that polypharmacy is associated with increased depressive symptoms,^11^ and so, as we had hypothesised, the increased rate of restarting antidepressant treatment is likely to represent an association with depression relapse.

We had also hypothesised that shorter durations of previous antidepressant treatment would be associated with an increased rate of restarting antidepressants and that longer treatment durations, having enabled the adequate treatment of depression previously, would attenuate the association between higher levels of polypharmacy and restarting antidepressants. However, conversely, the longer the previous antidepressant treatment duration, the higher the rate of restarting antidepressants. Although clinical trials in the general population have shown that participants are more likely to relapse from depression when they discontinue antidepressants compared with maintaining antidepressant treatment,^8^ the optimal treatment duration is unknown. A recent study investigating depression relapse following discontinuation of long-term antidepressant use (≥2 years) still found high rates of depression relapse compared with the control group who maintained treatment.^8^ Longer durations of antidepressant treatment have also been found to be associated with depression relapse in meta-analysis.^8^ Indeed, we found no evidence to suggest that longer periods of antidepressant treatment could reduce the risk of depression relapse following discontinuation in people with T2DM. However, the previous duration of antidepressant treatment could be a proxy for depression severity, with those participants with longer previous durations of treatment being more severely depressed and therefore more likely to subsequently relapse. This fits with our suggested explanation for the association between polypharmacy and restarting antidepressants, namely that the association may be caused by more severe depression in individuals with higher levels of polypharmacy. On the other hand, participants with shorter durations of previous antidepressant treatment may not have tolerated the antidepressant, and so may be less keen to restart similar treatments. Another potential explanation may be the misidentification of restarting antidepressant treatment. Individuals with longer previous durations of antidepressant treatment may have accumulated a back supply of medication. This could allow them to have a longer gap between prescriptions without discontinuing. This would mean that these individuals had not restarted treatment, but simply the continuation of the original course of treatment.

### Study strengths and limitations

With a sample size of 48 001, this study is over 100 times larger than the largest RCT investigating antidepressant relapse following discontinuation in any population.^8^ This has enabled us to more precisely model the relationship between restarting antidepressant treatment and a complex exposure, such as polypharmacy, using spline functions. The use of spline functions, as opposed to linear-based modelling, has enabled us to identify the minimum and maximum values for which the association with our outcomes was present (by fitting spline points within this range) and to identify the true size of the association compared with a baseline value of zero. This approach only reports associations that it identifies, therefore, there is no HR available for one concurrent medication or after 18 concurrent medications. In addition, the use of penalised-fit spline functions, as opposed to categorising the number of medications into clinically relevant groups (e.g. 1–4, 5–10), has allowed us to show that the rate of restarting antidepressants increases for each additional concurrent medication – a clinically relevant finding that would have been obscured by using broader categorisations.

Our outcome of restarting antidepressant treatment was intended as a potential marker of depression relapse. Almost all prescribing in UK primary care is electronic, and the vast majority of prescriptions are entered accurately on a patient's EHR. Thus, we have high confidence in the completeness and accuracy of restarting antidepressant treatment as our outcome variable. On the contrary, depression diagnoses, episodes or symptoms are known to be significantly under recoded,^24^ and therefore, would not be suitable to identify depression relapse. Restarting antidepressant treatment does not represent systematic assessment for depression as an outcome. Individuals experiencing depression relapse may or may not present to healthcare services, or still may choose not to restart antidepressants. However, restarting antidepressant treatment represents clinically identified depression, whereby the clinician and patient have identified a need to restart treatment. Further research would be beneficial to identify individuals experiencing relapse who do not restart antidepressant treatment.

It is also important to note that we were unable to directly account for previous depression severity, which may be associated with polypharmacy and longer previous durations of antidepressant treatment, and may increase the subsequent risk of relapse.

As we only included individuals who started antidepressant treatment with one of the most commonly prescribed antidepressants in the UK, we may have missed a subgroup of individuals with diabetic neuropathic pain, who may have been prescribed antidepressants such as duloxetine to treat both depression and pain together.

Furthermore, our study only included participants with depression whereas antidepressants may also be prescribed to treat anxiety.^25^ Further research is needed investigating antidepressant outcomes in individuals with comorbid anxiety and T2DM.

RCTs are the gold standard for evaluating medication outcomes and often have strict inclusion criteria thereby enabling case definition and outcome definition certainty. However, the strict inclusion criteria also often excludes complex populations, such as the participants in this study with comorbidities and concurrent polypharmacy.^5^ In addition, their use of predefined interventions and end-points prevent them from investigating real-world medications use, where the decision to discontinue or restart treatment is based to a large extent on patient/clinician preferences and behaviours. Our use of routinely collected EHR data enabled us to investigate real-world prescribing patterns, populations and prescribing outcomes.

### Implications

Polypharmacy and longer durations of previous antidepressant treatment combined are associated with up to 132% higher risk of restarting antidepressant treatment following discontinuation, in individuals with comorbid depression and T2DM. This may reflect an association with depression relapse. Further research is required to understand whether the association is with polypharmacy itself, confounding by indication of depression severity or multimorbidity. Further research is also required to understand whether there are key medications or conditions that drive the association. Enhanced support and monitoring when discontinuing antidepressants may be of benefit for individuals with T2DM and high levels of polypharmacy, particularly those who have been on antidepressants for longer durations, as these individuals may be at higher risk of relapse. Additionally, further research is required to establish optimum antidepressant treatment durations, including whether this differs across specific patient groups such as people with T2DM.

## Data Availability

Data are not publicly available in line with UK data protection laws, however, pseudonymised data are available following the appropriate ethical approvals from the UK CRPD.

## Supplementary material

For supplementary material accompanying this paper visit https://doi.org/10.1192/bjp.2022.160. click here to view supplementary material
