# Supplementary material for: Association between polypharmacy and depression relapse in individuals with comorbid depression and type 2 diabetes: a UK electronic health record study
Source: Br J Psychiatry. 2023 Mar;222(3):112–8. doi: 10.1192/bjp.2022.160 (PMC9929703; doi:10.1192/bjp.2022.160)
Supplement: Supplementary file 1 [file S000712502200160Xsup001.docx]

SUPPLEMENTARY DATA TABLES – Sensitivity analyses including participants the association between primary and secondary exposures and restarting antidepressant treatment

|  | **Sensitivity model 1A***  **HR (95% CI)** | **Sensitivity model 2A***  **HR (95% CI)** | **Sensitivity model 3A***  **HR (95% CI)** |
| --- | --- | --- | --- |
| **Number of medications**:**  **0**  **2**  **3**  **4**  **5**  **6**  **7**  **8**  **9**  **10**  **11**  **12**  **13**  **14**  **15**  **16**  **17**  **18** | Reference point  1.12 (0.84-1.50)  1.25 (0.90-1.75)  1.31 (0.96-1.79)  1.41 (1.03-1.93)  1.54 (1.12-2.11)  1.52 (1.11-2.08)  1.66 (1.21-2.27)  1.81 (1.31-2.49)  1.98 (1.43-2.74)  1.95 (1.40-2.72)  1.76 (1.24-2.49)  1.60 (1.10-2.34)  1.49 (0.97-2.30)  1.44 (0.83-2.51)  1.43 (0.66-3.11)  1.43 (0.46-4.42)  1.44 (0.30-7.02) | Reference category  1.10 (1.03-1.18)  1.22 (1.09-1.36)  1.33 (1.17-1.52)  1.45 (1.26-1.66)  1.55 (1.35-1.77)  1.63 (1.42-1.87)  1.73 (1.50-1.98)  1.82 (1.59-2.10)  1.91 (1.65-2.20)  1.97 (1.70-2.29)  2.02 (1.72-2.39)  2.07 (1.72-2.50)  2.13 (1.69-2.67)  2.19 (1.65-2.90)  2.25 (1.59-3.19)  2.32 (1.51-3.56)  2.39 (1.43-4.00) | Reference category  1.08 (1.01-1.15)  1.16 (1.04-1.30)  1.25 (1.09-1.42  1.33 (1.16-1.54)  1.40 (1.22-1.62)  1.46(1.27-1.69)  1.54 (1.33-1.77)  1.62 (1.34-1.87)  1.70 (1.46-1.97)  1.76 (1.51-2.06)  1.83 (1.55-2.17)  1.90 (1.57-2.31)  1.98 (1.57-2.50)  2.06 (1.55-2.74)  2.15 (1.51-3.05)  2.24 (1.46-3.44)  2.34 (1.39-3.92) |

* Sensitivity model 1A = redefining exposure to include repeat prescriptions only; Sensitivity model 2A = redefining outcome to limit observed follow-up to 6 months; Sensitivity model 3A = including participants with completed ethnicity only; all models adjusted for duration of previous antidepressant treatment, age, gender, ethnicity, calendar year and GP practice

**Medications prescribed simultaneous to the date of the last antidepressant prescription before discontinuation, excluding the antidepressant itself

|  | **Sensitivity model 2B***  **HR (95% CI)** | **Sensitivity model 3B***  **HR (95% CI)** |
| --- | --- | --- |
| **Duration of previous antidepressant treatment:**  **Early**  **NICE**  **WHO**  **Medium**  **Maintained** | Reference category  1.60 (1.51-1.70)  1.79 (1.68-1.92)  2.13 (2.03-2.23)  2.36 (2.25-2.48) | Reference category  1.69 (1.57-1.82)  1.85 (1.69-2.01)  2.13 (2.00-2.27)  2.49 (2.34-.2.64) |

* Sensitivity model 2B = redefining outcome to limit observed follow-up to 6 months; Sensitivity model 3B = including participants with completed ethnicity only; all models adjusted for age, gender, ethnicity, calendar year and GP practice
